# Cross-cultural perspectives on social difficulties and anxiety in youth: the role of family cultural values in Mexico and Italy

**DOI:** 10.3389/fpsyg.2025.1680963

**Published:** 2025-11-18

**Authors:** Ingrid Zugey Galán-Vera, Rachele Lievore, Ughetta Moscardino, Irene C. Mammarella

**Affiliations:** Department of Developmental and Social Psychology, University of Padova, Padua, Italy

**Keywords:** cross-cultural study, social difficulties, social anxiety, family cultural values, familism, traditional gender roles

## Abstract

**Introduction:**

Children and adolescents who experience social anxiety might face challenges in their social skills, with negative consequences for their quality of life. Contextual factors such as family cultural values may impact the relationship between these variables. In the present study, we aimed to investigate the association between social anxiety and social difficulties in Mexican and Italian samples, considering the possible influence of family cultural values (i.e., familism, traditional gender roles).

**Methods:**

The study involved 537 Mexican, and 541 Italian parents of children and adolescents aged between 6 and 18 years, who completed an online survey on their children’s skills and anxiety, and their own family values.

**Results:**

The results suggested that in both countries, social anxiety was positively linked to social difficulties. However, this association was attenuated by higher levels of familism while exacerbated by higher levels of traditional gender roles.

**Discussion:**

Psychoeducational programs should inform parents on the key role of family values in shaping children’s social and emotional functioning.

## 1 Introduction

Social skills are specific abilities that enable an individual to perform effectively in social tasks (Cavell, 1990). These skills include socio-cognitive and communication abilities, but also prosocial behaviors, like helping others, cooperating and sharing (Bierman et al., 2010; Bukowski et al., 2011). The development of social skills during childhood and adolescence is associated with better social adjustment, quality relationships with peers, and better academic performance (Kamper-DeMarco et al., 2020). When these abilities are not fully cultivated, they can lead to various social challenges, characterized by a range of internal and external behaviors that result in ineffective, minimal, or inappropriate social interactions (Achenbach et al., 2016; Conti-Ramsden and Botting, 2004), as well as greater social withdrawal (Biggs et al., 2012; Chiu et al., 2021). During childhood and adolescence, both individual variables related to psychological aspects (i.e., social anxiety), and contextual variables associated with the socio-cultural environment (i.e., family values), may have an impact on the optimal development of social skills. Several studies have investigated direct relationships among different variables involved in social skill development (Bierman et al., 2010; Cavell, 1990; She et al., 2023). However, the ways in which these relations may be shaped by external factors, especially when such phenomena coexist, remain understudied. Indeed, the development of youth’s social skills is shaped by a dynamic interplay between socio-emotional aspects, and contextual influences, including family environment and cultural norms. However, little research has explored the moderating role of contextual factors in the association between social difficulties and social anxiety.

As regards individual factors related to social skills, social anxiety can affect directly the development and performance of social skills, with direct consequences on the daily lives of individuals (Lacombe et al., 2024). Social anxiety is characterized by an intense fear of one or more social situations in which an individual may be exposed to possible scrutiny by others—a fear that is disproportionate to the actual threat (Stein and Stein, 2008). Consequently, social anxiety can negatively influence the development of social skills, often leading individuals to avoid social interactions. Anxiety may cause individuals to make negative misattributions about the others’ behavior, predetermining their own social responses (Kamper-DeMarco et al., 2020). At the same time, engaging in social interactions can trigger symptoms of social anxiety for children and adolescents who face difficulties with social skills (Beidel et al., 2014; Chiu et al., 2021; She et al., 2023). Some studies suggest that this condition may be present from the early stages of life (Wittchen and Fehm, 2003), with an estimate of 1–2% in children and 2% to 6% in adolescents (Aune et al., 2022; Spence et al., 2018). Children with high levels of social anxiety and deficits in social skills have also been observed to exhibit generalized and selective mutism (Cunningham et al., 2006), as well as lower peer acceptance and lower quality of friendships (Greco and Morris, 2005), low self-concept and somatic symptoms (Ginsburg et al., 1998; Przepiorka et al., 2021; Strahan, 2003). Among adolescents, those who experience social anxiety tend to exhibit deficits in socialization skills, which negatively impact interpersonal functioning (Alfano and Beidel, 2011).

Concerning contextual variables, those related to the family environment can directly influence the development and maintenance of social difficulties (Griffith et al., 2016; Yue et al., 2024). In particular, family cultural values are principles that act as guides, or patterns that family members follow, influencing their behaviors and social interactions (Blum-Kulka, 2012; Elsayed, 2024). In the context of parenting, caregivers choose their behavior from a range of available options, with their values playing a key role in guiding the selection and execution of these behaviors (Gamble and Modry-Mandell, 2008; Harwood et al., 1995). Among these values, familism refers to attitudes toward intra- and intergenerational support and commitment to family members, emphasizes attachment, loyalty, and obligation to the family, which prevails over individual interests (Cahill et al., 2021; Sabogal et al., 1987). Although there are no studies that have investigated the association between family cultural values and the possible outcomes on children’s social skills, some studies suggest that family cohesion can predict improvements in social problem solving and self-efficacy in children (Boyraz and Sayger, 2011; Leidy et al., 2010). Moreover, familism has been negatively associated with feelings of loneliness, depression, and physical symptoms, while being positively linked to school engagement and better self-concept (Corona et al., 2017; Stein and Stein, 2008). Another crucial family cultural value is traditional gender roles (TGR), which imply an unequal distribution of power and rights between men and women within the family. In this framework, husbands are typically viewed as the primary breadwinners and decision makers, while women are expected to take care of housework duties and childcare (Heise et al., 2019; Pinho and Gaunt, 2021). Gender role identification emerges through socialization during childhood and adolescence, as individuals learn their gender roles from parents and later from peers. It has been found that cultural differences in gender role identification are associated with social anxiety (Zentner et al., 2023).

Abundant evidence indicates that there is large variation in family values both within and across cultures, especially when the countries are in different continents, with unique geographical settings, cultural heritages, and societal structures. In this study, we compared Mexican and Italian families as they share both similarities and differences in their cultural values, as described below. The rationale for including these two specific countries lies in the fact that, although they are located on different continents, they share Latin roots and, consequently, cultural characteristics. One such characteristic is the central role of family relationships in children’s development, which often extends into adulthood, with frequent family interactions forming an integral part of daily life (Delvecchio et al., 2016; Ria Novianti and Islami, 2023). Mexico and Italy also share Catholicism as the predominant religion, which influences many practices within both family life and broader society (Butler, 2016; Vignoli and Salvini, 2014). Furthermore, although the official languages are different, they share significant linguistic similarities. Language, as a key aspect of culture, plays an essential role in shaping social interactions (Agha, 2006).

Despite Mexico and Italy share some commonalities, they also exhibit significant differences in cultural, economic, social, and historical aspects. In Latin America, high levels of cohesion and solidarity among extended families are common, with family support becoming the norm in most families (Cahn et al., 2018; Weymans, 2023). The case of Mexico is particular, since it is the only country in Latin America that shares a border with a developed country, and it has a culture more inclined to collectivism (Baeza et al., 2022; Barkin, 2019). In Mexico, family values are characterized by obedience to parents, respect over affection, and honor that should be maintained toward the family. Moreover, there is a predominance of traditional gender roles with respect to childcare, as mothers are the ones who generally fulfil this role, having more authoritarian and punitive tendencies with their children (Delgado-Herrera et al., 2024; Diaz-Guerrero, 2010; Ria Novianti and Islami, 2023). However, Mexican parents who have higher levels of familism also show a more supportive co-parental style, as do fathers who show higher levels of respect; on the contrary, high levels of sexism have been associated with a less supportive co-parental style (Lindsey, 2018). Although there is no evidence from studies in Mexican populations on the relationship of social skills, anxiety and family values, one study showed that familism in Mexican culture may promote better school and emotional adjustment in the interpersonal relationships of preschool children (Gamble and Modry-Mandell, 2008).

On the other hand, in Europe, and especially in Mediterranean countries (e.g., Italy), family cultural values are characterized by the primordial place of the family over other aspects of life - all members are expected to remain geographically and psychologically close to care for other members (Alesina and Giuliano, 2014; Boh et al., 2023). Despite this, Italy has an individualistic culture, it is one of the most important economies in the European Union, being among the first 15 in the world (Burton et al., 2021; Ghisellini and Ulgiati, 2020). Additionally, education has undergone transformation plans that include the creation of decentralized environments, the redesign of professional subjectivities, and the establishment of an evaluation process to measure the effectiveness of the system (Garelli, 2016; Grimaldi and Serpieri, 2014). Italian families preserve traditional gender roles, also because of the influence of the highest religious seat visible in today’s culture (Delvecchio et al., 2016). In parenting, the rules and restrictions that exist for children at an early age tend to create dependency on parents in adulthood, but during adolescence there is a more relaxed parental control and a parenting style characterized by negotiation and induction (Claes et al., 2018; Violato et al., 2017). Although research on the association between family cultural values and social skills is lacking in Italy, a recent study concluded that there is a relationship between parental cultural values and parenting practices, as well as internalized problems in children, for instance parental collectivism (i.e., parents values concerning their children’s autonomy and sense of belonging to a social group) was associated with higher expectations in children’s obligations (Bacchini et al., 2024).

To date, no studies have examined how contextual factors, such as family cultural values, may moderate the relationship between individual factors, like social anxiety and social skills, in children and adolescents across two geographically and culturally different countries (i.e., Mexico and Italy). Therefore, this study aims to explore whether it is the cultural similarities or differences between Mexico and Italy that weigh more heavily in understanding the socio-emotional development of children and adolescents in these countries. The study was conducted by investigating parents’ perspectives, as primary caregivers play a central role in shaping children’s socio-emotional experiences and in transmitting family cultural values that influence their development.

## 2 The present study

The aim of this study was to investigate the association between social skills, operationalized in this research as different levels of social difficulties, by considering the possible influence of both individual (i.e., social anxiety), and contextual factors (i.e., family cultural values), in a sample of Mexican and Italian children and adolescents aged 6 to 18 years. Exploring these relationships may contribute to establishing a stronger foundation for understanding how contextual factors influence the social development of youth. Considering different cultures allows us to grasp the extent of their impact and, importantly, including a country that does not belong to the Western, educated, industrialized, rich, and democratic (WEIRD) societies provide visibility to underrepresented and less-studied societies in current research (Wooliscroft and Ko, 2023).

The specific objectives were: (a) to detect possible differences in social difficulties, social anxiety and family cultural values (i.e., familism, TGR) between Mexican and Italian participants,

(b) to investigate the association between social difficulties and social anxiety, by considering some control variables, such as sex, children’s age and parental education, and to understand how contextual factors, such as familism and TGR, may moderate this association, and finally,

(c) to understand whether familism and TGR could moderate the relationship between social difficulties and social anxiety differently in Mexico and Italy.

Concerning our first aim about possible differences between the two countries, we expected that levels of social difficulties and anxiety would be higher in Mexico than in Italy, given the levels of poverty and social inequality (Cahn et al., 2018). Both familism and traditional gender roles might be greater in Mexico due to the structural characteristics of its current society, characterized by obedience to parents and traditional gender roles with respect to childcare (Hietanen and Pick, 2015), while Italy seems to hold a more individualistic perspective.

Regarding the second aim, considering previous studies (Alfano and Beidel, 2011; Beidel et al., 2014; Cunningham et al., 2006), we expected to find a significant positive relationship between social difficulties and social anxiety in the total sample, as it has previously been observed that social anxiety can directly impact social skills in children and adolescents. In addition, we expected social difficulties to be negatively associated with familism, acting as a protective factor against social impairments, and a positive relationship between social difficulties and TGR (Corona et al., 2017; Leidy et al., 2010; Lindsey, 2018; Stein and Stein, 2008). Due to the lack of studies on the moderators between social difficulties and social anxiety, a more exploratory approach has been adopted to draw hypotheses. It is worth assuming that familism could moderate the association between social difficulties and social anxiety, with higher levels of familism dampening the association between social difficulties and social anxiety (Gamble and Modry-Mandell, 2008). Conversely, TGR may act as a risk factor for the development of social anxiety associated with social difficulties (Diaz-Guerrero, 2010; Giordano et al., 2005; Lindsey, 2018).

In relation to the third aim, since there are no previous studies comparing the above-mentioned variables in Mexico and Italy, an exploratory approach has been adopted. However, considering the characteristics of both countries, we expect that familism and TGR may have a greater moderating effect in Mexico than in Italy (Alesina and Giuliano, 2014; Diaz-Guerrero, 2010; Giordano et al., 2005; Lindsey, 2018; Moral De La Rubia, 2013).

## 3 Materials and methods

### 3.1 Participants

The initial sample included 1274 Mexican and Italian fathers and mothers of children and adolescents between 6 and 18 years. Parents were asked to answer an online survey on their children’s traits and abilities, such as social difficulties, social anxiety, and family cultural values. The survey also included a socio-demographic questionnaire at the beginning, gathering information about the child (i.e., sex, date of birth, any medical or neurodevelopmental conditions). Moreover, in this initial part of the survey, parents were asked to provide information on the respondent’s role (mother, father), their date of birth, educational background, religion, marital status, number of children, and whether they reside in a rural (i.e., a zone with low population density and limited access to services) or urban area (i.e., a zone with high population density and wide access to services).

Participants who had a diagnosis of developmental disorders such as attention deficit and hyperactivity disorder were excluded (*n* = 80), as well as participants diagnosed with autism (*n* = 39), specific learning disorders (*n* = 43), intellectual disability (*n* = 5), in addition to those who had a medical condition (*n* = 29) that could directly affect their mental abilities, such as epilepsy. Children with neurodevelopmental disorders or epilepsy may exhibit socio-emotional developmental trajectories that differ from those of children without such diagnoses. Consequently, their inclusion could introduce a representation bias in the general population without diagnoses. The final sample consisted of 537 Mexican and 541 Italian parents of children aged between 6 and 18 years.

Table 1 presents descriptive data on the final sample divided by country, including information related to both children and their parents, such as age, sex, siblings, living area, parental education, parents’ marital status, and parents’ religious background.

**TABLE 1:** Characteristics of the final sample (*N* = 1078).

Variables	Mexico		Italy	
N	537		541	
Age *M* (SD)	12.40 (3.59)		12.11 (3.29)	
Gender (M:F)	280 (52.2%):257 (47.8%)		276 (51%):265 (49%)	
Siblings (yes/no)	236/301		276/265	
**Living area**				
Urban	489		414	
Rural	48		127	
**Raters**	**Mothers**	**Fathers**	**Mothers**	**Fathers**
N	353	184	336	205
Age *M* (SD)	40.21 (8.24)	41.05 (10.12)	44.27 (7.56)	47.66 (7.32)
**Parental education level**				
Primary school	15 (2.7%)	28 (5.2%)	3 (0.55%)	2 (0.36%)
Middle school	73 (13.5%)	81 (15%)	23 (4.2%)	71 (13.1%)
Secondary school	167 (31%)	145 (27%)	282 (52.1%)	275 (50.8%)
Bachelor’s degree	244 (45.4%)	241 (44.8%)	59 (10.9%)	45 (8.3%)
Master’s degree or more	38 (7%)	42 (7.8%)	174 (32.1%)	148 (27.3%)
**Marital status**				
Single	48 (8.9%)	48 (8.9%)	17 (3.1%)	12 (2.2%)
Married	370 (68.9%)	372 (69.2%)	436 (80.5%)	439 (81.1%)
Divorced	35 (6.5%)	33 (6.1%)	23 (4.2%)	24 (4.4%)
Widowed	7 (1.3%)	2 (0.37%)	2 (0.36%)	1 (0.18%)
Free union	77 (14.3%)	82 (15.2%)	63 (11.6%)	65 (12%)
**Religion**				
Catholic	364 (67.7%)	348 (64.8%)	413 (76.3%)	407 (75.2%)
Christian	70 (13%)	54 (10%)	16 (2.9%)	15 (2.7%)
Other religion	15 (3.2%)	13 (2.5%)	14 (2.7%)	10 (2%)
Religiously unaffiliated	88 (16.4%)	122 (22.7%)	98 (18.1%)	109 (20.1%)

*M*, mean; SD, standard deviation; Other religion: Jehovah’s Witness, Mormon, Muslim.

### 3.2 Procedure

The study was approved by the Ethics Committee of the University of Padova. The preparation of the survey took place in December 2023. Participants were recruited via Bilendi & respondi, an ISO-certified international survey company^1^. Individuals were randomly selected from a pool of respondents (i.e., online panel) based on the following inclusion criteria: (1) parents of children aged between 6 and 18 years without any neurological condition; (2) residence in Mexico or in Italy. Quota-based sampling ensured that the sample was representative of the respective country populations in terms of age, gender, and geographic region.

Participants were directed via a study link to the Qualtrics platform and provided informed consent prior to completing the survey, which was available throughout January 2024 and took about 20 min to complete. Participants were recruited based on the global quotas reported above (i.e., gender of their child, age of their child, and region of residence and children without any neurological condition). The company guarantees complete data collection, with no missing data.

### 3.3 Measures

#### 3.3.1 Social difficulties

The Social Responsiveness Scale, Second Edition (SRS-2; Constantino, 2013) is a 65-item questionnaire, which can be completed by parents or teachers, and measures social communication and repetitive and restrictive behaviors that are related to autistic traits in both diagnosed children and the general population. For this study, the parents’ version was used, including all 53 items of the four subscales related to social difficulties: social awareness (8 items), social cognition (12 items), social communication (22 items) and social motivation (11 items). Parents were asked to choose the response that best described their child’s behavior over the past 6 months. Each of the items was evaluated on a Likert scale ranging from 0 (“not true”) to 3 (“almost always true”). Some examples of the items are: “He/she has good self-confidence,” “He/she has difficulty making friends, even when trying his or her best.” Higher scores in the test indicated more social difficulties. Raw scores were considered in the analyses. We used the Spanish (Urbina, 2017) and the Italian adapted version (D’Ardia et al., 2021), which showed good psychometric properties in the normative sample (Mexico: Ω = 0.83; Italy: Ω = 0.85). The reliability was computed for the total Social Communication and Interaction (SCI) scale, which is composed of the four subscales, as is commonly done in previous studies (e.g., Constantino, 2021; Urbina, 2017; D’Ardia et al., 2021). In our sample, an excellent reliability was obtained for both countries in the total score we included (Mexico: Ω = 0.97, C.I. = 0.96–0.97; α = 0.96, C.I. = 0.96–0.97; Italy: Ω = 0.97, C.I. = 0.96–0.97; α = 0.96, C.I. = 0.95–0.96).

#### 3.3.2 Social anxiety

The Social Phobia and Anxiety Inventory for Children (SPAI-C; Beidel, 1996) is a 26-item questionnaire that evaluates social anxiety levels in youth. Children are asked to report how often they feel anxious in certain potentially anxiety-producing social situations (e.g., reading aloud in class, performing in a play, eating in the school cafeteria), and assesses physical and cognitive characteristics of social phobia as well as avoidance behaviors. The parents’ version created by Higa et al. (2006) is identical to the children’s one, except the stem of each item starts with e.g., “My child is afraid of being the center of attention” rather than “I’m afraid of being the center of attention” and it has been used in another studies (Beidel et al., 2021; Vigerland et al., 2016). Each of the 26 items is rated on a 3-point scale (1 = “never or hardly ever,” 2 = “sometimes,” 3 = “most of the time or always”) as was previously used in other studies (Cederlund and Öst, 2013; Higa et al., 2006). The maximum score is 78, with higher scores indicating a higher level of social anxiety and worries. The SPAI-C has good internal consistency and is significantly correlated with the self-report version for children (Gauer et al., 2005; Olivares et al., 2010) (α = 0.94) (α = 0.90) respectively. In our sample, an excellent reliability was obtained for both countries (Mexico: Ω = 0.97, C.I. = 0.96–0.97; α = 0.96, C.I. = 0.96–0.97; Italy: Ω = 0.97, C.I. = 0.96–0.97; α = 0.95, C.I. = 0.94–0.96).

#### 3.3.3 Family cultural values

The Mexican American Cultural Values scales for Adolescents and Adults (MACVS; Knight et al., 2010) is a 50-item scale designed for parents and adolescents to assess family cultural values. Although this scale was originally designed for the immigrant Mexican population, it has been used with other populations (Gülseven and Carlo, 2021; Zucker et al., 2018), since it does not refer to values that can only be found in the Mexican population, but rather includes items that express behaviors and values that can be found to a greater or lesser extent in families around the world. For this study, the 16 items related to the familism scale, and the 5 items related to the traditional gender roles (TGR) scale were considered. The parents were asked to read statements about what people may think or believe, and to choose their answers on a Likert-type scale ranging from 1 (“not at all”) to 5 (“completely”) points. Some examples of items are: “Parents should teach their children that the family always comes first” (familism), “Men should earn most of the money for the family so women can stay home and take care of the children and the home” (TGR). Higher scores on the scales correspond to higher levels of familism and traditional gender roles within the family. In our sample, an excellent reliability was obtained for familism in both countries (Mexico: Ω = 0.92, C.I. = 0.91–0.93; α = 0.92, C.I. = 0.91–0.93; Italy: Ω = 0.90, C.I. = 0.88–0.91; α = 0.90, C.I. = 0.88–0.91) and a very good reliability was obtained for traditional gender roles (Mexico: Ω = 0.86, C.I. = 0.84–0.88; α = 0.86, C.I. = 0.84–0.87; Italy: Ω = 0.84, C.I. = 0.81–0.86; α = 0.83, C.I. = 0.81–0.85).

### 3.4 Statistical approach

Mean (M), standard deviations (SD) and one-way ANOVAs were run as preliminary analyses considering social difficulties, social anxiety, familism and traditional gender roles to explore possible differences between the two countries (Mexico, Italy). Effect sizes using Cohen’s *d* were also computed. In addition, descriptive statistics of normality using the Shapiro–Wilk test, skewness and kurtosis values, and correlations among the main study variables were computed separately for each country to examine preliminary associations. Descriptive statistics and correlations are available in the Supplementary Materials (Supplementary Tables 1, 2).

Then, a hierarchical linear regression analysis was conducted with social difficulties as dependent variable. In the first step we included age, sex, and parents’ educational level as covariates; in the second step we added social anxiety; in the third step we added familism and traditional gender roles, then in the fourth step we included the interaction between social anxiety and familism, and the interaction between social anxiety and traditional gender roles. In the fifth step, country was considered as a third moderator to evaluate whether there were any differences in the association between social anxiety and social difficulties in Mexico and Italy. Finally, in the last step, three-way interactions were run to investigate possible group differences in the association between variables (social anxiety*familism*country; social anxiety*TGR*country). This method was adopted because it is the most suitable approach in line with our second and third research objectives, which firstly aim to understand how contextual factors, such as familism and TGR, may moderate the association between social difficulties and social anxiety, and, ultimately, to determine whether familism and TGR might differently moderate this relationship in Mexico and Italy.

The best-fitting step was selected using information-theoretic (I-T) approaches, considering the Akaike Information Criterion (AIC) (Burnham et al., 2011) and the adjusted R^2^ (adj R^2^) (Miles, 2005). The AIC serves as an estimate of prediction error, offering insight into the relative performance of statistical models on a specific dataset. AIC values and adjusted R^2^ (adj R^2^), were calculated for each step. The step with the lowest AIC aims to minimize the anticipated loss of information. The higher the adj R^2^, the better the model step. A simple slope analysis with Johnson-Neyman was then performed to analyze the effect of the predictors on the dependent variable at low (−1 SD), mean, and high (+1 SD) levels of the moderator.

Data were analysed using R version 4.2.2 (R Core Team, 2022). The following R packages were used: “lm.beta” to calculate the standardized coefficients (Behrendt, 2014), “stats” (R Core Team, 2022) to perform the comparisons and hierarchical linear regression, the AIC was calculated with “AICcmodavg” (Mazerolle, 2023) and “ggplot2” (Wickham, 2016) for the plots.

## 4 Results

### 4.1 Preliminary analyses

Descriptive statistics and statistical comparisons between the two countries are presented in Table 2.

**TABLE 2:** Descriptive statistics of the variables by country and statistical comparisons between the countries.

Variable	Country	*M*	SD	Minimum	Maximum	F (df)	*p*	Cohen’s d
Social difficulties	Mexico	47.8	22.9	6	114	72.84 (1)	**<0.001**	0.25
	Italy	36.3	21.1	0	104			
Social anxiety	Mexico	40.8	12.3	26	78	21.56 (1)	**<0.001**	0.14
	Italy	37.6	10.2	26	78			
Familism	Mexico	57.1	12.7	16	80	0.02 (1)	0.87	0.005
	Italy	57.0	10.0	20	80			
TGR	Mexico	13.2	5.6	5	25	24.58 (1)	**<0.001**	0.15
	Italy	11.6	4.8	5	25			

*M*, mean; SD, standard deviation; TGR, traditional gender roles. Bold indicates *p* significat values at <0.001.

### 4.2 Hierarchical regression analysis

**FIGURE 1:**
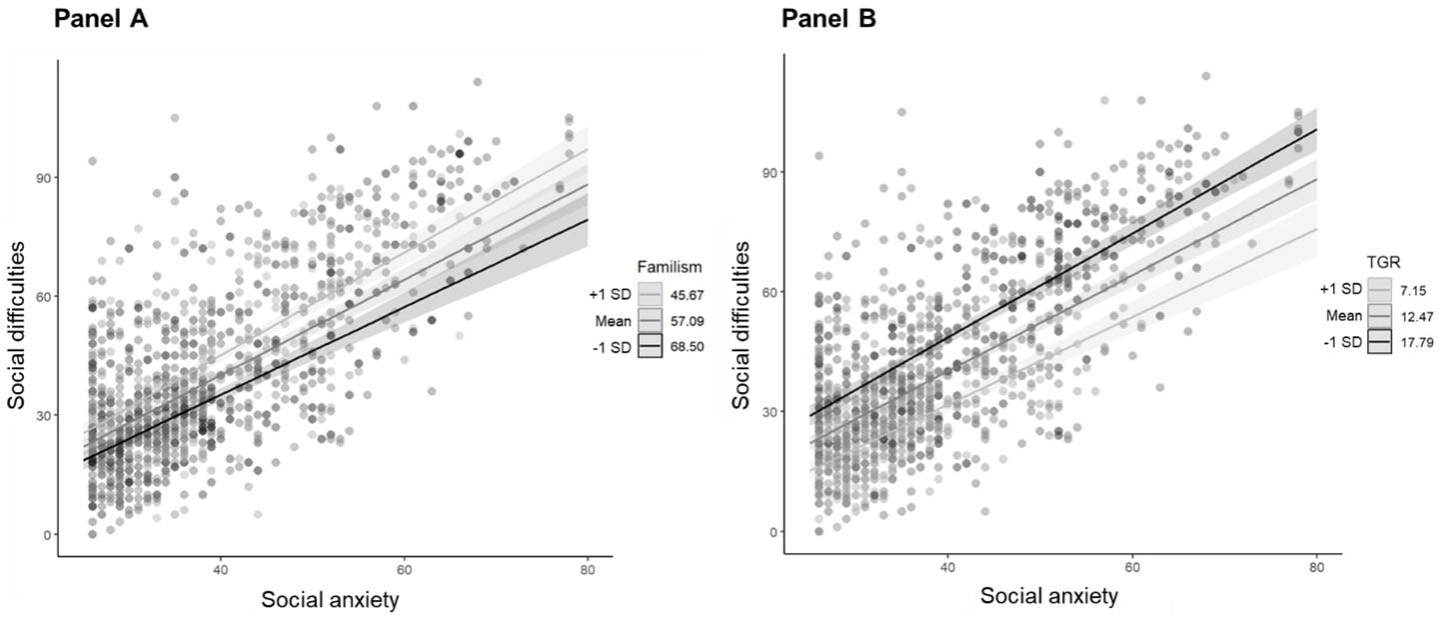
Significant interaction effects with social difficulties as dependent variable **(A)** social anxiety*familism; **(B)** social anxiety*TGR). Simple slope analyses are depicted. Bands represent 95% confidence intervals.

A series of linear regressions were tested using a hierarchical approach to investigate the association between social anxiety and social difficulties, also considering the moderating effect of familism and traditional gender roles. Moreover, country was entered in the fourth step to evaluate whether the quality of these relationships differed between Mexico and Italy. As shown in Table 3, the best-fitting model was Step 5: Social difficulties ∼ Age + Gender + Parental education + Social anxiety + Familism + TGR + Country + Social anxiety*Familism + Social anxiety*TGR + Social anxiety*Country (*F* = *189.0, p* < *0.001; AIC* = *8724.1, adj R^2^* = *0.63*). Statistically significant main effects resulted from the analyses, with parental education, *t* = −3.33, *p* = 0.009, social anxiety, *t* = 7.87, *p* = 0.001, TGR, *t* = 2.62, *p* = 0.009, and Country, *t* = 2.45, *p* = 0.01, predicting social difficulties. Moreover, two interaction effects were found to be statistically significant: social anxiety*familism, *t* = −2.26, *p* = 0.03; and social anxiety*TGR, *t* = 2.40, *p* = 0.01. However, the interaction between social anxiety and country was not significant. The simple slope analysis with Johnson-Neyman revealed that at low familism (−1 SD, 45.67), the slope of social anxiety was *B* = *1.30, SE* = *0.07, t* = *18.56, p* < *0.001*. At the mean level of familism (57.09), the slope was *B* = *1.21, SE* = *0.06, t* = *19.96, p* < *0.001*. At high familism (+ 1 SD, 68.50), the slope was *B* = *1.11, SE* = *0.08, t* = *14.34, p* < *0.001*. Instead, at low TGR (−1 SD, 7.15), the slope of social anxiety was *B* = *1.11, SE* = *0.08, t* = *13.99, p* < *0.001*. At the mean level of TGR (12.47), the slope was *B* = *1.21, SE* = *0.06, t* = *19.96, p* < *0.001*. At high TGR (+ 1 SD, 17.79), the slope was *B* = *1.31, SE* = *0.07, t* = *19.55, p* < *0.001*. Figure 1 presents the graphs of significant interaction effects of the best fitting model.

**TABLE 3:** Hierarchical regression analysis with social difficulties as dependent variable.

Regression model	Estimate coefficient	Standardized coefficient	SE	*t*	*p*	*F*	*p*	AIC	Adj R^2^
**Step 1**						7.01	<0.001	9787.9	0.01
Age	0.19	0.03	0.2	0.96	0.33				
Gender	−1.1	−0.02	1.38	−0.79	0.42				
**Parental education**	−**3.45**	−**0.13**	**0.78**	−**4.38**	**< 0.001**				
**Step 2**						285.4	<0.001	9029.4	0.51
Age	0.01	0.00	0.14	0.09	0.92				
Gender	−1.88	−0.04	0.97	−1.94	0.052				
**Parental education**	−**2.10**	−**0.08**	**0.55**	−**3.77**	**<0.001**				
**Social anxiety**	**1.41**	**0.70**	**0.04**	**33.16**	**<0.001**				
**Step 3**						298.6	<0.001	8755.1	0.62
Age	0.02	0.00	0.12	0.23	0.81				
Gender	−1.33	−0.02	0.85	−1.56	0.11				
**Parental education**	−**1.84**	−**0.07**	**0.49**	−**3.76**	**<0.001**				
**Social anxiety**	**1.21**	**0.60**	**0.03**	**31.27**	**<0.001**				
**Familism**	−**0.42**	−**0.21**	**0.04**	−**10.13**	**<0.001**				
**TGR**	**1.62**	**0.38**	**0.09**	**17.59**	**<0.001**				
**Step 4**						225.2	<0.001	8753.9	0.62
Age	0.02	0.00	0.12	0.2	0.83				
Gender	−1.25	−0.02	0.85	−1.46	0.14				
**Parental education**	−**1.92**	−**0.07**	**0.49**	−**3.91**	**<0.001**				
**Social anxiety**	**1.43**	**0.72**	**0.18**	**7.71**	**<0.001**				
Familism	−0.13	−0.06	0.14	−0.91	0.36				
**TGR**	**1.02**	**0.23**	**0.31**	**3.21**	**<0.001**				
Social anxiety*familism	−**0.008**	−**0.27**	**0.003**	−**2.04**	**<0.001**				
Social anxiety*TGR	**0.015**	−**21**	**0.007**	**1.99**	**<0.001**				
**Step 5**						189.0	<0.001	8724.1	0.63
Age	0.003	0.00	0.12	0.02	0.98				
Gender	−1.21	−0.02	0.84	−1.43	0.15				
**Parental education**	−**1.62**	−**0.06**	**0.49**	−**3.33**	**0.009**				
**Social anxiety**	**1.45**	**0.73**	**0.18**	**7.87**	**<0.001**				
Familism	−0.09	−0.04	0.15	−0.65	0.51				
**TGR**	**0.82**	**0.19**	**0.32**	**2.62**	**0.009**				
**Country**	**7.67**	**0.16**	**3.12**	**2.45**	**0.01**				
Social anxiety*familism	−**0.008**	−**0.30**	**0.004**	−**2.26**	**0.03**				
Social anxiety*TGR	**0.02**	**0.25**	**0.008**	**2.40**	**0.01**				
Social anxiety*country	−0.07	−0.06	0.08	−0.90	0.37				
**Step 6**						135.4	<0.001	8727.2	0.63
Age	0.004	0.00	0.12	0.03	0.96				
Gender	−1.19	−0.02	0.84	−1.41	0.15				
**Parental education**	−**1.58**	−**0.06**	**0.48**	−**3.24**	**<0.001**				
**Social anxiety**	**1.56**	**0.78**	**0.33**	**4.6**	**<0.001**				
Familism	0.02	0.01	0.24	0.1	0.91				
Traditional gender roles	0.34	0.07	0.48	0.7	0.47				
Country	10.36	0.22	16.12	0.64	0.52				
Social anxiety*familism	−0.01	−0.42	0.006	−1.91	0.05				
Social anxiety*TGR	**0.02**	**0.36**	**0.01**	**2.18**	**0.02**				
Social anxiety*country	−0.24	−0.23	0.4	−0.6	0.54				
Familism*country	−0.25	−0.32	0.3	−0.8	0.40				
TGR*country	0.97	0.32	0.64	1.5	0.13				
Social anxiety*familism*country	0.006	0.38	0.007	0.86	0.38				
Social anxiety*TGR*country	−0.01	−0.26	0.01	1.07	0.28				

TGR, traditional gender roles. Bold indicates *p* significat values at <0.001.

## 5 Discussion

The aim of this cross-cultural study was to examine the relationship between social difficulties and social anxiety, while considering the potential influence of family cultural values – specifically, familism and traditional gender roles - by comparing two culturally distinct yet similar countries (i.e., Mexico, Italy). In fact, most research has focused on understanding different types of interactions between personal factors (Przepiorka et al., 2021), while less is known about how contextual factors may be affecting those interactions. One of the strengths of this study lies in the implementation of a cross-cultural approach with a large sample size.

In relation to the first aim, which sought to detect possible differences across countries in social difficulties, social anxiety and family cultural values (i.e., familism, TGR), we observed a significant difference in the levels of social difficulties, social anxiety and traditional gender roles, being higher in the Mexican sample with a medium effect size, which is to be expected, given the sociocultural context. This may be due to the fact that in Mexico, social anxiety is one of the most common anxiety disorders (Castaños-Cervantes and Vélez-Agosto, 2020). Also, as Mexico is a developing country, the levels of poverty and social inequality are high, as well as the lack of education and effective health systems, which leads to the fact that children and adolescents in Mexico are exposed to various factors such as violence and family dysfunction that increase the risk of developing mental health problems (Casas-Muñoz et al., 2024) in comparison with Italy, in which there is greater social stability and less exposure to violence (Ghisellini and Ulgiati, 2020). In addition, traditional gender roles prevail in the general culture of Mexico due to the fact that these patterns of behavior continue to be transmitted from generation to generation (Hietanen and Pick, 2015). On the other hand, with respect to familism, no significant differences were found between the two countries, suggesting that, despite their differences, Mexico and Italy share the same family-centered importance. Although familism is generally considered aa a cultural value characteristic of Latin American culture and, for this reason, has been less frequently studied in other cultural contexts, our results indicate that the differences are not significant. This finding highlights the need for further research on the topic, as familism may extend beyond the Latin American context. It is also worth noting that Mexico and Italy share Latin roots, one of whose defining features is a strong sense of family closeness and connection. This cultural similarity may help explain the absence of significant differences observed in our study.

As regards the second aim concerning the association between social difficulties and social anxiety, a medium positive size effect was found, meaning that higher levels of social anxiety are associated with greater social difficulties. These results are congruent with the findings obtained in previous studies (Beidel et al., 2014; Bowers et al., 2020; Chiu et al., 2021; Habibi Asgarabad et al., 2023; She et al., 2023). Indeed, previous results revealed that high levels of social anxiety in children are associated with social difficulties, such as selective mutism (Cunningham et al., 2006), lower peer acceptance, such as less quality of friendships (Greco and Morris, 2005), low self-concept and more somatic symptoms (Przepiorka et al., 2021). Moreover, in adolescents, social anxiety is related to challenges with socialization skills, interpersonal functioning and initiating and maintaining relationships (Alfano and Beidel, 2011). Another important finding of the current study is the negative association between parental education (e.g., high school, master degree) and social difficulties: higher levels of parental education were associated with fewer social difficulties in children, consistent with previous results (Kajastus et al., 2023). A possible interpretation is that parents with higher levels of education tend to have more positive parenting attitudes and feel that their parenting is less stressful because they have more tools and a larger support network at their disposal. Indeed, parents with higher levels of education might take a more proactive role in deciding how much time and money they dedicate to resources and activities that support their children’s development (Griffith et al., 2016; Hosokawa and Katsura, 2018). However, the majority of parents in our sample possessed educational levels slightly above the national averages in both countries, with a relatively homogeneous distribution of education across participants. Therefore, these results should be further examined to determine whether they remain consistent in samples with greater variability in educational levels, and to assess whether the observed effect persists under more heterogeneous conditions.

Additionally, concerning how familism and traditional gender roles may moderate the association between social difficulties and social anxiety, our findings revealed two significant interaction effects. First, the impact of social anxiety on social difficulties decreased as familism levels increased, supporting previous findings obtained with different populations (Corona et al., 2017; Leidy et al., 2010; Stein and Stein, 2008). These results might imply that high levels of familism may act as a protective factor against the development of an interconnection between children’s social anxiety and social difficulties. Indeed, familism has been shown to act against different risk factors such as emotional maladjustment (Gamble and Modry-Mandell, 2008) and externalizing problems (Germán et al., 2009). Familism may provide emotional support to children and adolescents, fostering their social skills development and creating a strong sense of belonging, all of which help mitigate the impact of social anxiety on social difficulties (Stein et al., 2015; Zhao et al., 2022). Besides making it easier to navigate social situations without fear or avoidance, family support and collective coping strategies might offer a protective buffer against social challenges (Pinkerton and Dolan, 2007). This confirms our hypothesis about the positive influence of familism on the association between social anxiety and social difficulties.

Second, our analyses indicated that higher levels of TGR amplified the detrimental effect of social anxiety on social difficulties. Although there are no previous studies in which the same variables were studied, it has been found that cultural differences in gender role identification are associated with the presence of social anxiety depending on the importance given to masculinity and femininity in each culture (Zentner et al., 2023). Moreover, TGR can act as a risk factor in suicidal ideation (Coleman, 2015), risky sexual behavior (Shearer et al., 2005), self-harm (Straiton et al., 2013) and partner violence (Conzemius et al., 2021), all of them being associated with social challenges (Calati et al., 2019) and emotional maladjustment (Carey and Richards, 2014; Laye-Gindhu and Schonert-Reichl, 2005). TGR can act as a risk factor in the relationship between social anxiety and social difficulties by intensifying the expectations associated with gender-specific behaviors. For instance, TGR often expect boys to be confident and suppress emotional vulnerability, and emphasize empathy, agreeableness and social connectedness for girls (Fischer and Manstead, 2000; Kaufman, 2005; Löffler and Greitemeyer, 2023). However, when people perceive themselves as not meeting these expectations, they may feel pressure to conform to these roles, leading to heightened anxiety, further contributing to social withdrawal or difficulties (Arcand et al., 2020). These results highlight the influence that the values instilled by parents can have on children’s development and the need to further explore other possible effects.

Our final aim was related to understand whether these patterns of associations differed across Mexico and Italy. No differences emerged between the two countries. As previously mentioned, this similarity stem from the fact that, although Mexico and Italy are geographically distant countries, they share some characteristics like the Latin roots, the religion and a strong emphasis on the family as a core social institution. In fact, cohesion, solidarity and closeness to the family is an important aspect in both cultures (Alesina and Giuliano, 2014; Diaz-Guerrero, 2010; Reher, 2004), although familism has been assumed as a predominant value of the Latin American culture (Stein et al., 2015). Indeed, as discussed above, our findings revealed no significant differences in familism between Mexico and Italy. Regarding TGR, although Mexican parents reported higher levels as compared to the Italian ones, Mexico and Italy also share similarities that may prevent a differential effect on the relationship between social anxiety and social difficulties. In fact, in both countries, mothers are more in charge of housework and childcare, while fathers are more authoritarian, protective and the breadwinners of the family (Delgado-Herrera et al., 2024; Delvecchio et al., 2016; Ria Novianti and Islami, 2023). These results have allowed us to observe that family cultural values held similar effects on the association between social anxiety and social difficulties in Mexico and Italy, even though they do not share such a close historical relationship.

Although our study offers novel insights into the role of family values in the association between social anxiety and social difficulties in children and adolescents from different cultural backgrounds, some limitations need to be considered when interpreting the results. First, the study relies on a survey in which parents are asked to report on their children’s skills and behaviors. This approach may lead to overestimation, underestimation, or misinterpretation of these behaviors, influenced by the level of attention parents pay to them, their personal parenting style, or the sociocultural context in which they reside. Consequently, the survey results are based on parental perceptions, with all the biases that this entails, such as social desirability. Future studies would benefit from the inclusion of multiple reports provided by both the children and the teachers as well, in order to obtain a more comprehensive perspective. Additionally, it is important to emphasize that these results pertain to neurotypical populations. Therefore, it is possible that the findings might differ in populations with neurodevelopmental diagnoses, which were excluded from the sample. This exclusion should be considered when interpreting the generalizability of the results. Second, given the nature of an online survey, we cannot ensure that parents answered the questions accurately or with the required attention, that the instructions were clear to them, or that each item was read carefully. Furthermore, parents’ educational level was rather homogeneous which implies the possibility that some populations with lower levels of education are not being represented in the study, additionally, we do not consider differences in the marital status of the parents. Third, we did not consider within-country variations, since families were recruited in different regions across both countries. To address such nuances, future studies may involve different subgroups (e.g., immigrant/ethnic minority families) or regions (e.g., North-South, urban-rural) to ascertain whether family cultural values and their effects on the social anxiety-social difficulties link may vary in these populations. Future research should explicitly address intranational diversity, examining how regional and contextual differences, such as variations between North and South, urban and rural areas, or among different ethnic or socioeconomic groups, might shape family cultural values. Understanding these nuances could clarify whether the associations between cultural values and children’s social difficulties and social anxiety are consistent across different subpopulations within a country, thereby enhancing the cross-cultural applicability and depth of such findings. Relatedly, further research on the generalizability of our findings to countries with more individualistic cultural orientations and less emphasis on familism and TGR is warranted.

Our results may have practical implications for educational settings, since they allow us to observe the importance of the values and education that children and adolescents receive from their families, as these can directly influence their development and adaptation to the social environment. They also suggest that the close and supportive relationship children receive during these stages of life, can protect them from developing future relational problems. On the other hand, despite having some progress in gender equality, our findings suggest that there are still traditional patterns that negatively affect the new generations. Therefore, psychoeducational intervention programs could be implemented in which these issues are addressed and in which parents can learn new perspectives and obtain new tools to educate their children. Educational programs can also be implemented with children and adolescents to help them to strengthen new and more constructive values from an early age.

## 6 Conclusion

In conclusion, social difficulties and social anxiety may have a significant positive relationship, with higher levels of social anxiety linked to greater social challenges in neurotypical children and adolescents. Moreover, the developmental environment of these children and adolescents can play a role in their psychosocial adjustment. Specifically, family cultural values can moderate the relationship between social anxiety and social difficulties, where familism serves as a protective factor, while TGR becomes a risk factor.

## Data Availability

The raw data supporting the conclusions of this article will be made available by the authors, without undue reservation.

## Footnotes

1
https://www.bilendi.es/
