# Supplementary material for: Cross-cultural perspectives on social difficulties and anxiety in youth: the role of family cultural values in Mexico and Italy
Source: Front Psychol. 2025 Nov 18;16:1680963. doi: 10.3389/fpsyg.2025.1680963 (PMC12671049; doi:10.3389/fpsyg.2025.1680963)
Supplement: Supplementary file 1 [file Table_1.docx]

**Supplementary material**

**Table S1.** Descriptive statistics (Skewness, Kurtosis, Shapiro-Wilk test) of the Mexican and Italian samples.

| **Statistics** | **Social difficulties** | **Social anxiety** | **Familism** | **Traditional gender roles** |
| --- | --- | --- | --- | --- |
| **Mexico** |  |  |  |  |
| Valid | 537 | 537 | 537 | 537 |
| Skewness | .58 | .81 | -.41 | .45 |
| Std. Error of Skewness | .10 | .10 | .10 | .10 |
| Kurtosis | -.52 | -.11 | .36 | -.78 |
| Std. Error of Kurtosis | .21 | .21 | .21 | .21 |
| Shapiro-Wilk | .95 | .92 | .97 | .94 |
| P-value of Shapiro-Wilk | < .001 | < .001 | < .001 | < .001 |
| **Italy** |  |  |  |  |
| Valid | 541 | 541 | 541 | 541 |
| Skewness | .78 | 1.01 | -.03 | .62 |
| Std. Error of Skewness | .10 | .10 | .10 | .10 |
| Kurtosis | -.06 | .50 | -.20 | -.46 |
| Std. Error of Kurtosis | .21 | .21 | .21 | .21 |
| Shapiro-Wilk | .94 | .90 | .98 | .94 |
| P-value of Shapiro-Wilk | < .001 | < .001 | < .001 | < .001 |

**Table S2.** Spearman’s correlations between variables in the Mexican (lower diagonal) and Italian (upper diagonal) samples.

| **Variables** | **Social difficulties** | **Social anxiety** | **Familism** | **Traditional gender roles** |
| --- | --- | --- | --- | --- |
| **Social difficulties** | - | .62  < .001 | -.16  < .001 | .37  < .001 |
| **Social anxiety** | .63  < .001 | - | -.11  .01 | .15  < .001 |
| **Familism** | .05  < .001 | .09  < .001 | - | .22  < .001 |
| **Traditional gender roles** | .41  < .001 | .14  < .001 | .48  < .001 | - |
